# Spontaneous regression of transverse colon cancer with high-frequency microsatellite instability: a case report and literature review

**DOI:** 10.1186/s12957-018-1552-x

**Published:** 2019-01-15

**Authors:** Nozomi Karakuchi, Manabu Shimomura, Kazuhiro Toyota, Takao Hinoi, Hideki Yamamoto, Seiji Sadamoto, Koichi Mandai, Hiroyuki Egi, Hideki Ohdan, Tadateru Takahashi

**Affiliations:** 1 Department of Surgery, National Hospital Organization Higashihiroshima Medical Center, 513, Jike, Saijyo-cho, Higashihiroshima, Hiroshima, 739-0041 Japan; 2 Department of Surgery, National Hospital Organization Kure Medical Center and Chugoku Cancer Center, Hiroshima, Japan; 3 Department of Clinical Laboratory, National Hospital Organization Kure Medical Center and Chugoku Cancer Center, Hiroshima, Japan; 4 Department of Pathology, National Hospital Organization Higashihiroshima Medical Center, Hiroshima, Japan; 5 Department of Gastrointestinal and Transplant Surgery, Applied Life Sciences, Institute of Biomedical and Health Sciences, Hiroshima University, Hiroshima, Japan

**Keywords:** Colorectal cancer, Spontaneous regression, Microsatellite instability

## Abstract

**Background:**

Spontaneous regression (SR) of colorectal cancer (CRC) is extremely rare, and only few cases have been reported. Although it is not yet clarified, a plausible mechanism for SR of CRC is an immunological event.

**Case presentation:**

In this report, we present the case of SR of primary CRC in a 78-year-old man. Preoperative colonoscopy was performed, and a type 2 tumor measuring 30 mm in diameter in the transverse colon was detected. The biopsy revealed a poorly differentiated adenocarcinoma. Colectomy was performed 2 months after initial colonoscopy. During the surgery, only a 10-mm ulcer harboring a polypoid lesion measuring 8.5 mm was detected in the resected tissue; no other masses or carcinoma cells were seen on histological examination. Afterwards, the biopsy specimens were reanalyzed, and immunohistological analysis verified this as adenocarcinoma with stroma-infiltrating lymphocytes. Further analysis revealed a loss of two mismatch repair proteins, suggesting sporadic high-frequency microsatellite instability (MSI-H).

**Conclusion:**

According to previous literature, a common site of SR in CRC is the proximal colon, which is a feature of MSI-H CRC. However, our report showed a rare case of SR of CRC, which was in the transverse colon, with MSI-H present. This report indicates a relationship between immunological features of MSI-H and the occurrence of SR of CRC. A better understanding of this phenomenon and the mechanisms involved will have significant preventive and therapeutic implications for CRC, including anti-PD-1 immune checkpoint inhibitor therapy.

## Background

Spontaneous regression (SR) of malignant tumor is defined as their partial or complete disappearance in the absence of all treatment or in the presence of treatment that is considered inadequate to exert a significant influence on neoplastic disease [1, 2]. Although the detailed mechanism of SR has not been fully understood yet, an immunological event is reported as one of the possible causes of SR.

SR of colorectal cancer (CRC) is known to be extremely rare, accounting for less than 2% of all the SR cases [3]. Herein, we report a rare case of SR of transverse colon cancer in a 78-year-old man. We conducted immunostaining and found that the expression levels of the mismatch repair proteins (MMRs) were decreased, indicating that this tumor was a CRC with high-frequency microsatellite instability (MSI-H). Recent studies have reported the effectiveness of an anti-programmed cell death 1 (PD-1) antibody treatment for MSI-H CRC, because of its immunological characteristics [4]. In this paper, we summarized all the similar reported cases and reviewed the possible interactions between SR and MSI-H CRC.

## Case presentation

The patient was a 78-year-old man who had consulted the physician for paroxysmal atrial fibrillation (pAf), chronic heart failure, and chronic renal failure. Anti-coagulant therapy was administered to the patient for pAf. At a follow-up examination, the patient complained of tarry stool. The patient had no family history of cancer.

**Fig. 1:**
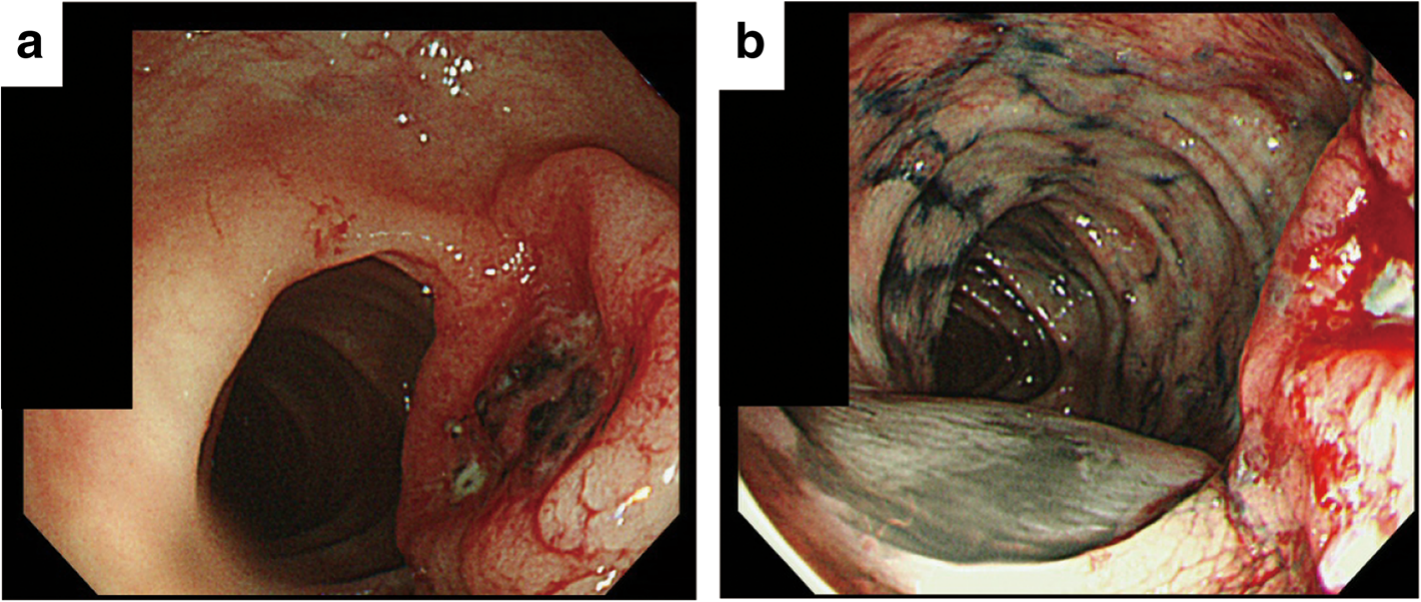
**a**, **b** Colonoscopy shows a type 2 tumor in the transverse colon measuring 30 × 30 mm. Marking was performed near the tumor for future surgical resection

A colonoscopy was performed and revealed a type 2 tumor in the transverse colon measuring 30 × 30 mm (Fig. 1a). Marking was performed by injecting a black dye into the submucosal layer, near the tumor, for future surgical resection (Fig. 1b). Biopsy specimens from the tumor suggested a poorly differentiated adenocarcinoma (Fig. 3a, b). Moreover, laboratory examinations revealed no remarkable abnormality: the carcinoembryonic antigen (CEA) and carbohydrate antigen 19-9 (CA19-9) levels were 3.1 ng/ml (< 5.0) and 3.4 U/ml (< 37), respectively. A computed tomography (CT) scan revealed wall thickening, which was the basis for diagnosing the lesion, as the tumor invaded the muscularis propria (T2); moreover, there was no evidence of lung, liver, or lymph node metastases. The clinical diagnosis was T2N0M0, stage I according to the TNM classification (UICC 8th edition).

**Fig. 2:**
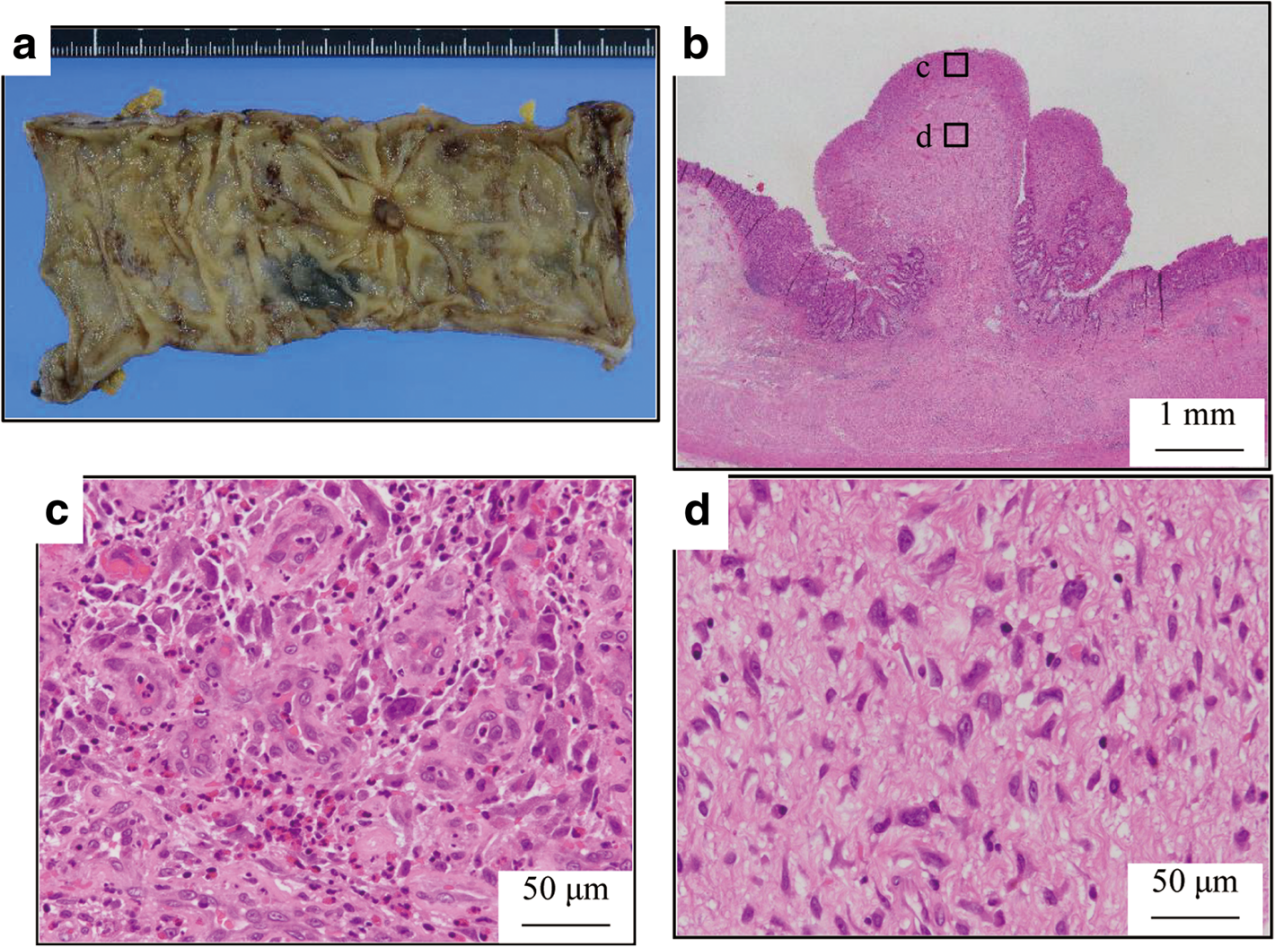
The resected specimen reveals a 10-mm ulcer with plicate concentration, which has an 8.5-mm-sized polypoid lesion in the center (**a**). Histological examination shows marked nonspecific granulated tissue where the epithelium sloughed and fibrillization under the mucous membrane (**b**). No cancer cells were found in the scar tissue (**c**, **d**)

**Fig. 3:**
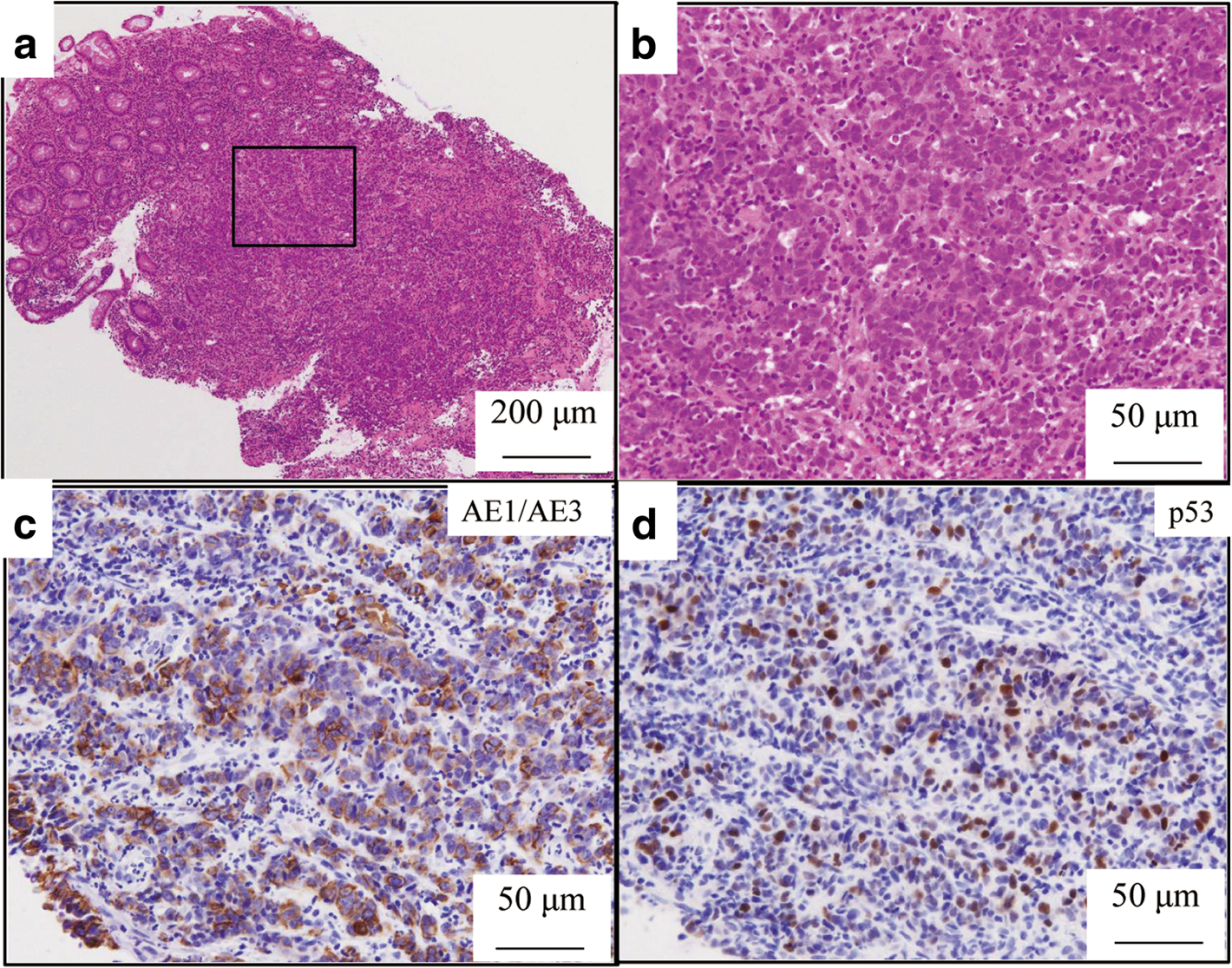
Hematoxylin-eosin staining shows a poorly differentiated adenocarcinoma with tumor-infiltrating lymphocytes (TILs) in the tumor stroma (**a**, **b**). Immunohistological examination shows strong positivity for AE1/AE3 (**c**) and positivity for p53 (**d**) in the tumor; therefore, it was proven to be an adenocarcinoma

**Fig. 4:**
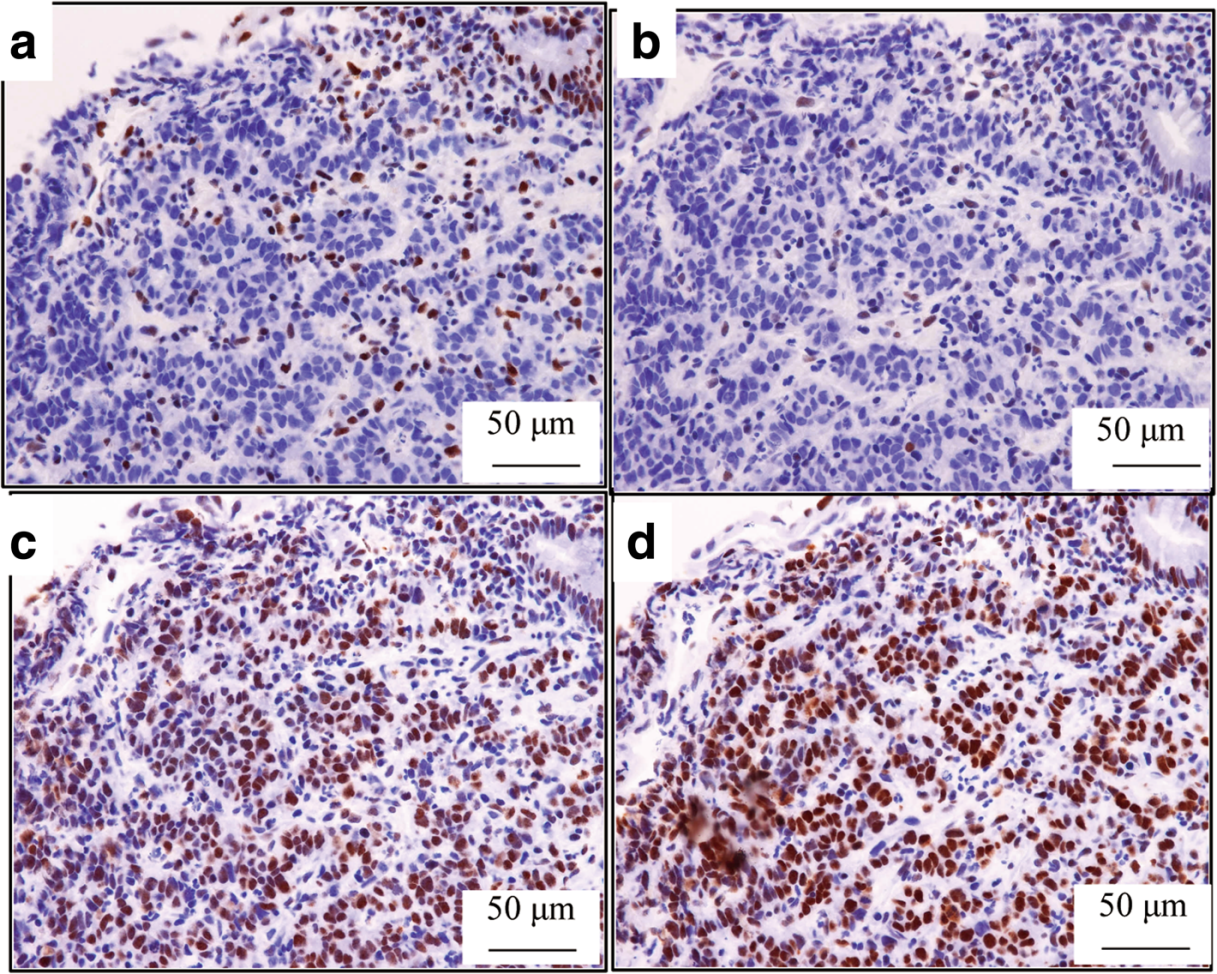
Immunohistochemical examination of mismatch repair proteins shows lack of MLH1 (**a**) and PMS2 (**b**) expression in tumor nuclei, in contrast to the positive staining for MSH2 (**c**) and MSH6 (**d**). This evidence indicates that the tumor was an MSI-H CRC due to MLH1 functional abnormality. Intact nuclear staining in the stromal lymphocytes or adjacent normal epithelia was used as the internal positive control for mismatch repair proteins

Laparoscopy-assisted colectomy was carried out 2 months after the initial colonoscopy. The patient did not receive any alternative medications, such as supplements, vitamins, and immunotherapy. We resected the colon, including the marking made during colonoscopy. The resected specimen revealed a 10-mm ulcer with a polypoid lesion of 8.5 mm in the center (Fig. 2a), but there was no type 2 tumor. The formalin-fixed specimen was cut into 3–5 mm slices. Histological examination demonstrated a marked nonspecific granulation of tissue, indicating fibrillization under the mucous membrane and sloughing off of the epithelium (Fig. 2b). Moreover, no cancer cells were found in the scar tissue (Fig. 2c, d). The dissected lymph nodes also did not show the presence of cancer cells. We used immunohistological staining to further evaluate the biopsy specimen. The findings showed that the tumor cells were strongly positive for AE1/AE3 (Fig. 3c) and positive for p53 (Fig. 3d), indicating that it was an adenocarcinoma. These findings suggested SR of colon cancer. Hematoxylin-eosin staining showed poorly differentiated adenocarcinoma, with tumor-infiltrating lymphocytes (TILs) in the tumor stroma. Based on these pathological features including poorly differentiated adenocarcinoma and TILs and the tumor location in the proximal colon, we suspected MSI-H CRC (Fig. 3a, b). Immunohistochemical examination of MMRs showed a lack of MLH1 (Fig. 4a) and PMS2 (Fig. 4b) expression in tumor nuclei, in contrast to the positive staining for MSH2 (Fig. 4c) and MSH6 (Fig. 4d). The loss of PMS2 expression is known to be followed by the loss of MLH1 expression due to heterodimerization, suggesting that this case was attributed to functional abnormality of MLH1, which is required to generate high level of MSI [5]. Lynch syndrome was not suspected because of its absence in the family and past histories; therefore, we reached a diagnosis of sporadic MSI-H CRC.

The postoperative course was uneventful. The patient did not receive adjuvant chemotherapy or other anticancer treatment. Six months after surgery, a periodic colonoscopy was performed, and no abnormal findings were seen. Subsequently, there was no evidence of cancer recurrence noted after 1 year.

## Discussion

To the best of our knowledge, this is the first report of SR of primary CRC that is proven to be with MSI-H. This is an important report, since it suggests a relationship between immunological features of MSI-H and the occurrence of SR of CRC.

SR of cancer is reported to occur in approximately one in 60,000 to 100,000 people [6]. Challis et al. have reported 741 cases of SR of cancer from the year 1900 to 1987, wherein there were 99 cases of renal cancer, 92 of malignant melanoma, and 68 of malignant lymphoma. There were 18 cases of colorectal cancer, which was only 2% of all the SR cases [3]. Considering a large number of CRC cases, CRC is considered to less likely undergo SR [7].

The mechanisms of SR are still unclear. The postulated mechanisms affecting SR of cancer in general include immunological, endocrine, metabolic, surgical treatment of the primary tumor and postoperative events; elimination of a carcinogen; elimination of an antigen; angiogenesis inhibition; tumor necrosis; oncogenes; growth factors; cytokines; genetic and epigenetic; induction of benign differentiation; apoptosis; and psychological factors [8]. As for SR of CRC, the following factors were suggested: (1) ischemia by mass increase, (2) mechanical stimulation by the bowel peristaltic movement, (3) circulation imperfection by the torsion or the traction of the mass, and (4) participation of the biopsy [9]. In addition, Cole et al. have suggested that immunological reactions may cause SR by forming specific antibodies in response to the antigenic tumor cells [10]. Recently, Chida et al. have reported the possible interaction between SR of CRC and immune-mediated antitumor response, based on the presence of TILs in the biopsy specimens [11].

There are two predominant forms of genomic instability characterized by CRC: chromosome instability (CIN) and microsatellite instability (MSI). The MSI-H CRCs account for approximately 15% of all CRCs and result from dysfunction of the DNA MMR system [12]. Moreover, MSI-H CRCs show peculiar clinical-pathological features, such as prevalence in the proximal colon and histological characteristic types, which include poorly differentiated adenocarcinoma or mucinous adenocarcinoma. Although polymerase chain reaction is the standard for assessing DNA MMR competency, immunohistochemical staining for MMR proteins (e.g., MLH1, MSH2, MSH6, and PMS2) has recently emerged as a valuable complementary technique [13]. A previous study showed the loss of MMR protein expression as determined by immunohistological staining to be highly concordant with DNA-based MSI testing, with good sensitivity (> 90%) and excellent specificity (100%) [14]. Additionally, previous clinical studies suggested that patients with MSI-H stage II or III colorectal tumors do not appear to benefit from fluoropyrimidine-based adjuvant chemotherapy [15]. MSI-H CRCs also tend to be associated with high numbers of TILs, which make them optimal candidates for immune therapy [16]. In fact, recent studies have reported the effectiveness of anti PD-1 antibody for MSI-H CRCs, because of their immunological characteristics [17].

In the case report, MSI-H was strongly suspected to be present because of the clinicopathological findings (right sided, poorly differentiated adenocarcinoma, TILs) and the MMR proteins deficiency. As for a superficial CRC with pedunculated lesions, one of the causes of SR is mechanical stimulation. However, SR due to mechanical stimulation is less likely to occur in CRC with muscular invasion, such as in the presented case. We, therefore, tried to review similar SR cases with muscular invasion to understand the mechanisms of SR and their relationships with MSI-H. A literature search was conducted using the PubMed and Ichushi databases to obtain English and Japanese literature describing SR of primary CRC with muscular invasion, and only six cases, including our case, had been reported since 2000 (Table 1). The keywords used in the search were “spontaneous regression” and “colorectal cancer.” In this literature search, there were five men and two women, with a median age of 74 years, who were found to have SR of primary CRC. The median tumor size was 26 mm, and the median duration of SR was 2.5 months. The operation was performed in six of the seven cases, and there were no cases of recurrence. In six of these seven cases, the tumor was located on the right side of the colon, and only one case was rectal in origin. Poorly differentiated adenocarcinoma was observed in two of the cases, and the other cases were of the differentiated type [7–9, 15, 19]. In this review, a tendency of the tumors to appear in the proximal colon, which is one of the features of MSI-H CRC, was observed. This finding was not conclusive because the other clinicopathological features, including histological type, were not indicative of an MSI-H phenotype. In future, further examinations are needed to determine the MSI status in other similar cases.

**Table 1:** Reported cases of spontaneous regression of advanced colorectal cancer (2000–2017)

Author (year)	Age	Sex	Primary site	Size (mm)	Histology	Duration (month)	Prognosis (month)	Operation
Sakamoto (2009) [18]	80	M	Rectum	25	tub1	3	ND	+
Shimizu (2010) [10]	80	M	Transverse	25	tub2	7	64 (alive)	−
Nakashima (2009) [9]	76	F	Ascending	20	tub1	2	18 (alive)	+
Sekiguchi (2013) [15]	69	F	Ascending	20	tub2	1.5	ND	+
Kihara (2015) [19]	64	M	Transverse	30	tub2	1.5	12 (alive)	+
Chida (2017) [11]	80	M	Transverse	30	por	1	12 (alive)	+
Present case (2017)	70	M	Transverse	30	por	2	11 (alive)	+

In our case, we do not know how or why the cancer was eliminated during the development. One of the possible hypotheses was that the cancer cells were recognized as antigens during investigations such as colonoscopy and biopsy.

## Conclusions

We have presented a rare case of SR of transverse colon cancer with MSI-H. SR of CRC has a tendency to occur in the proximal colon, suggesting a possible interaction between SR of CRC and immunological features of MSI-H. Since a better understanding of this phenomenon and the mechanisms involved will have significant preventive and therapeutic implications for CRC, including anti-PD-1 immune checkpoint inhibitor therapy, further investigation is needed including testing of MSI status for such cases.

## Abbreviations

CA19-9: Carbohydrate antigen 19-9
CEA: Carcinoembryonic antigen
CIN: Chromosome instability
CRC: Colorectal cancer
CT: Computed tomography
MMRs: Major mismatch repair proteins
MSI-H: High-frequency microsatellite instability
pAf: Paroxysmal atrial fibrillation
SR: Spontaneous regression
TILs: Tumor-infiltrating lymphocytes
